# Potential Risk of Virus Carryover by Fabrics of Personal Protective Gowns

**DOI:** 10.3389/fpubh.2019.00121

**Published:** 2019-05-22

**Authors:** Iyoko Katoh, Fuminori Tanabe, Hirotake Kasai, Kohji Moriishi, Noriko Shimasaki, Katsuaki Shinohara, Yukiko Uchida, Tomoko Koshiba, Soichi Arakawa, Michiko Morimoto

**Affiliations:** ^1^Oral Health Science Research Center, Kanagawa Dental University, Yokosuka, Japan; ^2^Faculty of Medicine, University of Yamanashi, Chuo, Japan; ^3^Influenza Virus Research Center, National Institute of Infectious Diseases, Tokyo, Japan; ^4^Division of Biosafety Control and Research, National Institute of Infectious Diseases, Tokyo, Japan; ^5^Faculty of Health and Welfare, Takasaki University of Health and Welfare, Takasaki, Japan; ^6^Faculty of Fashion Science, Bunka Gakuen University, Tokyo, Japan; ^7^Sanda City Hospital, Sanda, Japan; ^8^Faculty of Health and Welfare Science, Okayama Prefectural University, Soja, Japan

**Keywords:** personal protective equipment, infection, surgical gown, fabric, health care workers, virus, water repellency, sliding angle

## Abstract

Personal protective gowns and coveralls are classified based on barrier efficiency that validates protection from fluid penetration under certain pressures. Materials standardized in this system have been found suitable for emergency medical practices confronting highly contagious diseases. Nevertheless, adhesion of blood, and body fluids from virus-infected patients to the surface of protective clothing still imposes a risk of pathogen transmission in the process of doffing, or undressing. We performed a small-scale experiment to test the possibility of infectious virus carryover on the surface of different fabrics used in commercially available protective gowns. Application of a lentivirus vector that expresses green fluorescent protein allowed easy monitoring of infectious viral loads on fabrics. Results indicate that fabrics of level-3 surgical gowns serve better to reduce virus transmission compared to fabrics of chemical protective clothing with the same or higher barrier efficiency. Analysis of sliding angles provided indexes of fluid repellency, which were inversely related to virus carryover potentials. Droplets of infectious body fluids may easily roll off fabrics with water-repellent finishing. Thus, virus carryover is a measurable risk factor to be considered for better choice of personal protective clothing.

## Introduction

Personal protective equipment (PPE) is essential to guard healthcare workers (HCW) in emergency departments and in wards with highly contagious patients. Gowns and coveralls, as other components of PPE, are designed to prevent transmission of pathogens contained in the blood and body fluids of patients (1, 2). Presently, PPE is classified by barrier efficiency that certifies protection from penetration of fluids, bacteria, and bacteriophage under defined pressures. However, selection of isolation gowns involves consideration of various attributes including classification standards, guidelines, and effectiveness (3). As inferred from the Ebola and SARS outbreaks, availability in the facility, tolerance to distress in the protective gowns, and conditions of patients are also important issues (4). In fact, overheating was found as a major concern in HCW who worked with PPE (5, 6).

Importantly, the process of removing PPE has the highest risk of contact transfer of viruses from the PPE surface to the skin of HCW (4, 7, 8). Many reports have highlighted careful instructions of removal procedures to HCW (6, 8). Furthermore, application of repellent finish was thought to reduce the risk of body fluid carryover by the gowns (1). In contrast to the emphasis given on barrier efficiency, which is categorized as levels 1–4 by standards such as ANSI/AAMI PB70 and ISO16603/16604, the impact of fluid repellency has been poorly documented. We designed a small-scale biological experiment to detect the infectious viral loads on the surfaces of commercially available PPE fabrics. In this challenging study, all experiments were conducted with priority for qualitative index, but not for “evaluation” or “judgment” on statistical analyses.

## Materials and Methods

### Fabrics

The sources and features of the fabrics examined in this study are listed in Table 1. Product S is a single layer of polyethylene, and is usually used as a cover on nurse uniforms. H and J are sterile disposable surgical gowns that meet the AAMI level 3 standard. Coveralls V, M, and C are non-sterile disposable protective clothing that meet ISO standards of liquid and bacteriophage barrier function. Fabric pieces (7 mm square) were cut out using sterilized tools in the safety cabinet. Most PPE fabrics of this dimension provided a sufficient area for droplet attachment, and could be kept flat throughout the experiment. Fabrics of unsterilized gowns (S, V, M, and C) were exposed to UV-light for 10 s on each side.

**Table 1:** Fabrics tested in this study.

**Fabric**	**Standards: barrier efficiency**	**Materials**	**Repellent finish**	**Suggested application**	**Manufacturer, product**
S	ANSI AAMI^a^ Level-1	Polyethylene		Protective cover	Saraya^f^, Plastic gown
H	ANSI AAMI^a^ Level-3	Non-woven polypropyrene, spunbond/meltblown/spunbond	**√**	Surgical gown	Hogy Medical^f^, Salem
J	AAMI^a^ Level-3	Non-woven polypropyrene, spunbond/meltblown/spunbond, 5 layers	**√**	Surgical gown	JMS^f^, Opegown III
V	EN ISO 22610a^b^ Class-1, ISO16603^c^ Class-3	Flash spun high-density polyethylene		Chemical protective garment	DuPont, Tyvek (400)
M	EN ISO22610^b^ Class-4, ISO 16604^d^ Class-4	Laminated fabric (Polypropylene+ microporous film)		Pharmaceutical manufacturing, agriculture and veterinary services	XINYUAN^g^, Metec Plus-T
C	EN 14126 Type 4B^e^ ISO16603^c^ Class-6, ISO16604^d^ Class-6	Fabric V with polymer coating		Protection against chemical and biological hazards	DuPont, Tychem C

a *Standard for isolation gowns*.

b *Bacterial penetration breakthrough time (Class-1, ≤ 15 min) (Class-4, >45 min)*.

c *Resistance to penetration of blood and body fluids (Class-3, >3.5 kPa) (Class-6, >20 kPa)*.

d *Resistance to penetration of blood borne pathogens (bacteriophage φX174) (Class-4, >7 kPa) (Class-6, >20 kPa)*.

e *Protective clothing against radioactive contamination*.

f *Japan*.

g *China*.

### Green Fluorescence Protein (GFP)-Lentivirus

Experiments were performed in biosafety level-2 facilities under the protocols approved by Institutional Biosafety Committee of University of Yamanashi. For experimental safety and easy counting of infected cells, we used a self-inactivating lentiviral empty vector that produces *Aequorea coerulescens* GFP in infected cells. The virus is referred to as GFP-lentivirus in this study. Lenti-X 293T cells were transfected with pLVSIN-acGFP1-C1 plasmid and the Lentiviral High Titer Packaging Mix (Clontech). At 48 h, the culture supernatant was harvested, centrifuged, and further clarified by passing through a membrane filter (pore size 0.45 μm). Aliquots of the virus suspension (~5 × 10^5^ infectious units/mL) were frozen at −80°C until use.

### Small-Scale Virus Carryover Experiment

A graphic summary of this experiment is shown in Supplementary Figure 1. HeLa cells used as virus recipient cells were plated (8 × 10^4^ cells/well, 96 mm^2^) in a glass-bottomed 24-well plate (SensoPlate, Greiner) 24 h before virus infection. A droplet (40 μL) of GFP-lentivirus-containing fluid (culture medium, D-MEM, supplemented with 10% fetal bovine serum and antibiotics) was placed on a plastic plate and immediately a fabric piece was placed on the droplet so that the surface was in contact with the droplet for 1 min. The fabric was then carefully lifted with a fine-tipped tweezer. The fluid attached to the fabric was retrieved in culture medium (200 μL), and the residual medium on the fabric was precipitated in a 1.5 mL centrifuge tube by spinning at 1,000 rpm for 10 s. HeLa cells were incubated with the retrieved virus suspension containing 8 μg/mL of Polybrene (Santa Cruz Biotechnology). At 44 h post-infection, cells were incubated with fresh medium containing Hoechst 33342 (2 μg/mL) for an additional 1 h.

### Microscopic Observation and Cell Counting

Three fields (~10 mm^2^) of blue (nuclei stained with Hoechst 33342) and green (cells infected with GFP-lentivirus) fluorescence were imaged for each well using the Keyence BZ-9000 fluorescence microscope. Cells were counted using the BZ-II *Dynamic Cell Count* Ver. 1.01 program in the BZ-9000 Analysis Software.

### Analysis of Cytotoxicity and Anti-virus Activity

The surface of the fabric piece was incubated with complete culture medium (200 μL) in the wells of a 24-well plate for 24 h at 37°C. To analyze the cytotoxicity of materials eluted from the fabric surface, HeLa cells were cultured with the fabric-incubated medium for 24 h, and the amount of ATP was measured using an ATP assay kit (Abcam, ab83355). We also tested the fabric-incubated medium for GFP-lentivirus infectivity.

### Measurement of Sliding Angles

We analyzed the sliding angles of fluid droplets on the fabrics as described previously (9). A droplet (50 μL) of culture medium containing 10% serum was placed on the fabric (148 × 210 mm) fixed to a tilting stage (DMo-501SA, Kyowa, Japan). The stage was inclined (2°/s) until the droplet began to slide, or roll off. Sliding angle was defined here as the stage angle at 0.5 s (1° incline) before the droplet began to slide. The droplet volume required for measurement was determined be 50 μL using fabrics H and M.

## Results

### Virus Carryover by the Fabrics

**Figure 1:**
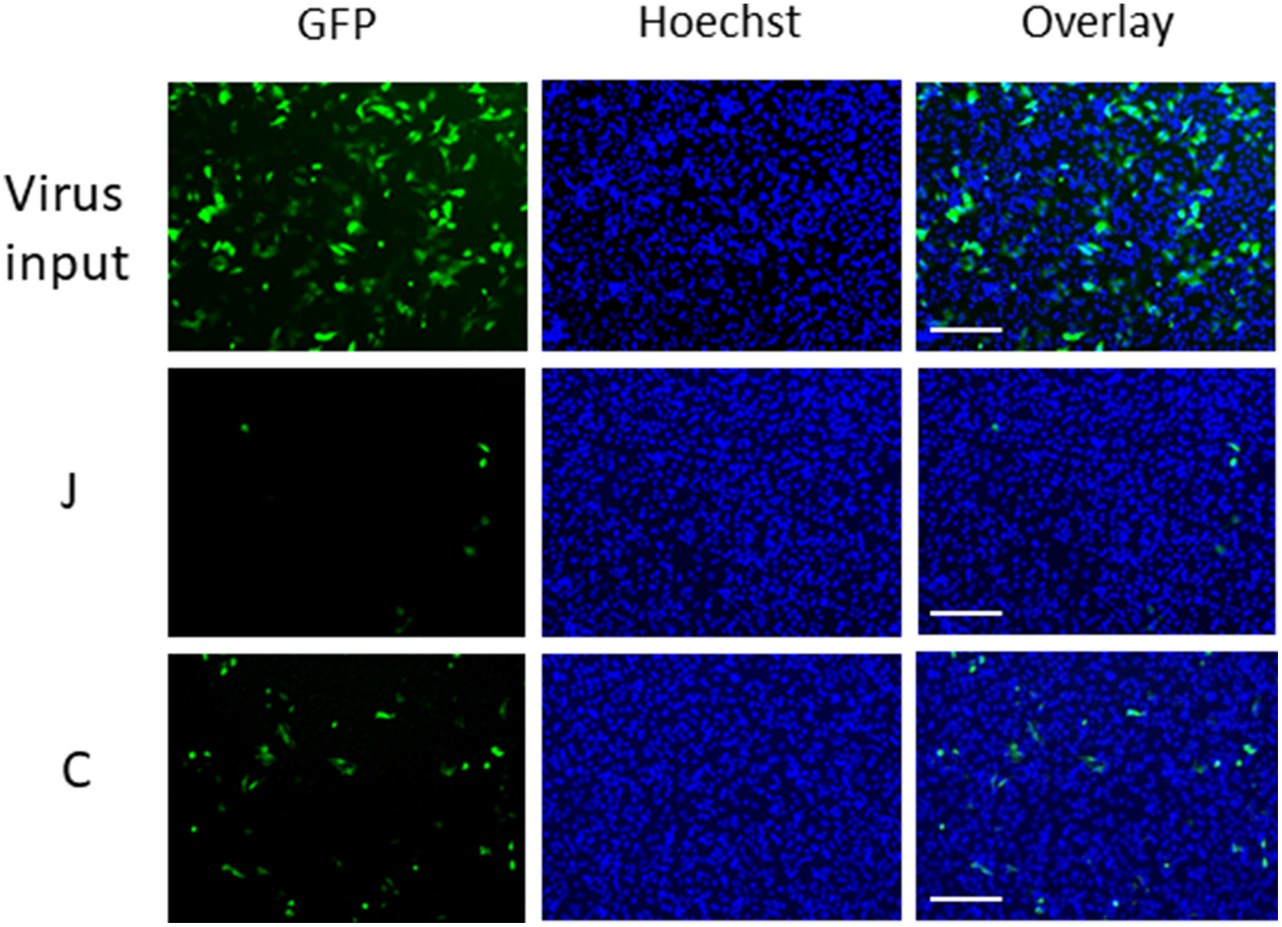
Microscopic observation of cells infected with GFP-lentivirus transferred by fabrics J and C. Each panel corresponds to a 1/9 part of each view field (10 mm^2^) in which GFP-positive cells and Hoechst-stained cells were counted. Scale bars indicate 200 μm.

We applied GFP-lentivirus produced by pLVSIN-acGFP1-C1 in this study, because the virus particles infect cells only once, and do not spread through the cultures. When GFP-lentivirus particles in a 40 μL droplet of culture medium (with 10% serum) infected HeLa cells directly, ~2,300 cells (~30%) were found GFP-positive in a background of 7700 Hoechst-stained nuclei in areas of 10 mm^2^ (Figure 1, upper panels). In the virus carryover experiments, fabric pieces were placed on GFP-lentivirus-containing droplets so that the fabric surfaces came into contact with the fluid (Supplementary Figure 1). The fluid retrieved from each fabric piece was used to infect HeLa cells and GFP-expressing cells were assessed. Interestingly, the GFP-positive cell numbers varied from one fabric to another. For example, an increased number of GFP-positive cells were detected with fabric C (bottom) than with fabric J (middle).

**Figure 2:**
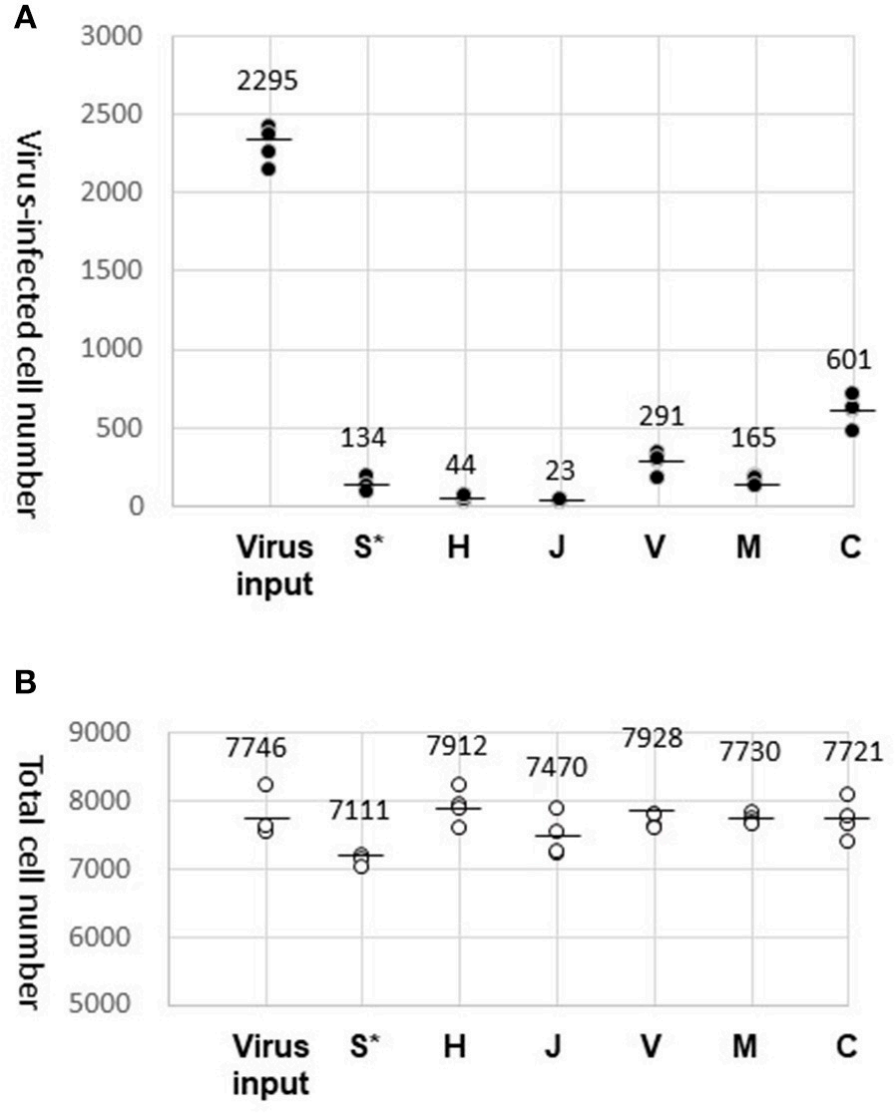
Results of virus carryover experiments. **(A)** Virus-infected cell number. GFP-positive cell counts (average of three different view fields of 10 mm^2^) were obtained for four wells and plotted (dark dots). The mean of the four well counts is indicated by a bar. In addition to the virus carryover by the test fabrics (S, H, J, V, M, and C), the entire virus load (Virus input) in the droplet (40 μL) were also examined. (*After attachment of the droplet, fabric S was directly submerged in the well of HeLa cell culture). **(B)** Total cell number. Hoechst-stained cells were counted and plotted in the same way as in **(A)** (circles).

We counted the cells with blue (Hoechst 33342) and green (GFP) fluorescence using the cell counting program (Figure 2). The data obtained by our biological experiments in small sample size are presented by scatterplots, but not by bar graphs (10). The entire cell counts did not differ significantly among the tested fabrics, H, J, V, M, and C, and were similar to the counts with the virus-containing 40 μL droplet (virus input) (Figure 2B). A small fraction (1–2%) of the GFP-lentivirus in the droplet was transferred by fabrics H and J (Figure 2A). Surprisingly, more efficient virus transfer (5–30%) was observed with V, M, and C. The virus-containing fluid was the most adhesive to fabric C with the strongest barrier efficiency (Table 1). These results suggest that virus carryover potentials increase in the order: H, J < M < V, C. We performed this experiment in quadruplicate and repeated three times to obtain comparable results.

Fabric S, a soft polyethylene material, was easily crumpled and could not tolerate the virus recovery process. Alternatively, fabric S was directly submerged in the medium of HeLa cell culture after the virus attachment. Results obtained by this procedure were plotted in the S^*^ column for reference only (Figure 2A). The Hoechst-stained cell counts were decreased by ~10%, where a certain amount of virus transfer by fabric S was evident by the detection of GFP-positive cells.

### Absence of Anti-viral or Cytotoxic Materials on the Fabric Surface

**Figure 3:**
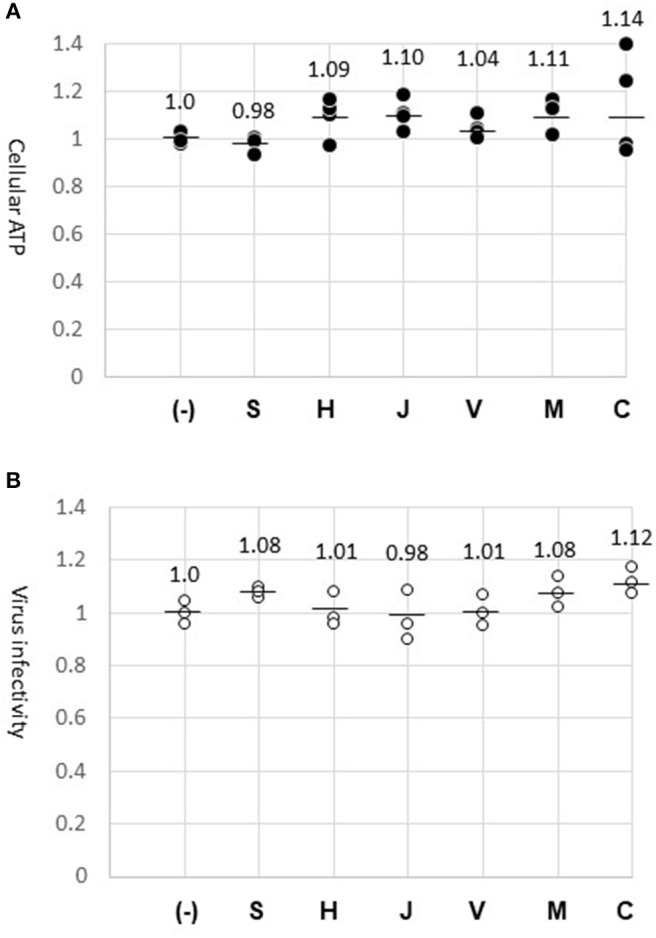
Absence of cytotoxic or anti-virus material on fabric surfaces. **(A)** Cellular ATP. Cells were incubated for 24 h with the media previously exposed to the surface of fabrics (S, H, J, V, M, and C) or the normal medium (–). ATP amount in the cell lysate was measured in four wells (dark dots) and indicated in relation to the control experiment (1.0). The mean of four measures is shown by a bar. **(B)** GFP-lentivirus stock was diluted 1:10 in the fabric-exposed medium for infecting HeLa cells. At 45 h, virus-infected cells were counted in three wells. The virus-infected cell numbers are indicated (circles) in relation to the control experiment (1.0).

We considered the possibility that chemicals eluted from the fabric might have interfered with the GFP-lentivirus infection. The fabric surface was exposed to culture medium for 24 h to allow soluble materials to dissolve into the medium. A constant amount of GFP-lentivirus was diluted in the fabric-exposed medium to infect HeLa cells. At the end of the infection period (45 h), GFP-positive cells were counted. As a result, the virus infected cell number was not altered by the fabrics tested, confirming that the virus infection process was not affected by materials on the fabrics (Figure 3B). In parallel, cells were cultured in the same medium without the virus, and were analyzed for ATP amounts. Cellular ATP production was not significantly altered (Figure 3A), suggesting that the fabrics had no cytotoxic effect. Thus, the differences in GFP-lentivirus transfer among the fabrics (Figure 2A) were not caused by materials eluted from the fabrics. These experiments with the fabric-elution media were done two times without causing a significant deviation.

### Measurement of Sliding Angles

We attempted to evaluate fluid-repellency as opposed to fluid-adhesion, using an alternative technique other than the above-described biological method. Sliding angles, also termed “shedding angles” and “roll-off angles,” indicate the water-repellent properties of textiles (9, 11).

**Figure 4:**
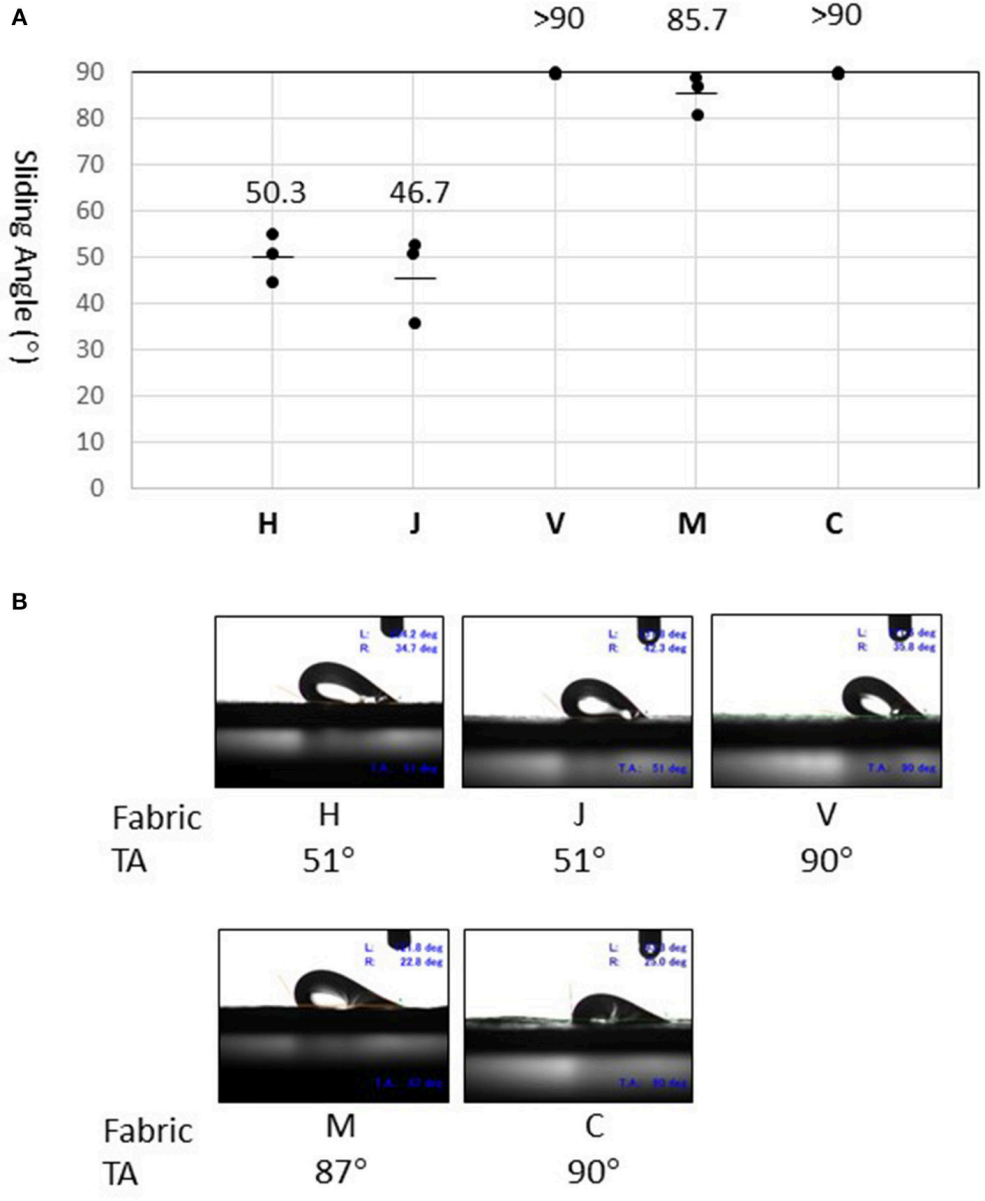
Measurement of sliding angles. **(A)** Sliding angle is here defined as the stage angle at 0.5 second (corresponding to tilt angle of 1°) before the droplet began to slide. Values (°) obtained in three experiments are shown by dots. The mean of three measures is indicated as a bar for fabrics H, J, and M. On fabric V and C, the droplet did not slide even when the tilt angle (TA) reached 90° (as indicated by >90). **(B)** Snapshots of the droplet at the moment of sliding angle measurement. Experiments were recorded using a movie camera fixed to the stage. The monitor also displayed the tilt angle (TA), advancing contact angle (left, L), and receding contact angle (right, R).

Sliding angles determined for fabrics H and J (50.3° and 46.7°, respectively) were significantly lower than that of fabric M (87.5°) (Figure 4A). Surprisingly, the fluid droplet did not roll-off fabric V or C, even when the stage was tilted to 90° (Figure 4B). Thus, the test fabrics showed stronger fluid-repellency in the order: H, J > M > V, C. This result is consistent with the fact that fabrics H and J have a water-repellent finish, whereas the others do not (Table 1). Furthermore, fluid repellencies were inversely related to virus carryover potentials (Figure 2).

## Discussion

Fabrics of personal protective gowns were tested for the possibility of GFP-lentivirus carryover. Adhesion of virus-containing fluid was found to vary from one fabric to another. Interestingly, surgical gown fabrics of class-3 (H, J), as determined by the standards of ANSI AMI, showed significantly decreased virus adherence property compared to chemical protective coveralls with the same (V) or higher barrier efficiencies (M, C). Fluorocarbon-based finishes are commonly used for surgical operation gowns made of fabrics H and J (1). These types of gowns may offer good choices for HCW who care for patients in epidemic/pandemic incidences, under the conditions where excessive pressures against the gowns are not anticipated.

SARS and MERS coronaviruses survive on dry surfaces for days or weeks depending on the experimental methods and environments (12). Influenza viruses have relatively shorter survival times, but can remain infectious at least for hours (12). The GFP-lentivirus used here represents HIV and is related to HTLV, both of which are blood borne viruses. Virus adhesion to PPE surfaces thus imposes a considerable threat to HCW, especially in doffing procedures when they leave contagious wards. Even ordinary hospital gowns were found as carriers of bacteria and viruses (13, 14).

Substantial refinement is necessary to develop more feasible and reliable assay methods suitable for statistical analyses. Furthermore, we noted workable modifications to the present system: (i) composition of test fluids (blood, other body fluids, or their substitutes); (ii) adhesion time; (iii) virus retention time on the fabrics; (iv) the use of different types of viruses, etc.

Sliding angles serve as good indicators of water-repellency in superhydrophobic textiles (9, 11). The technique is simple and more reliable than other conventional techniques such as contact angle determination, and may be applicable to PPE fabric evaluation. It is emphasized that the sliding angle measurements for the five different PPE fabrics directly corresponded to their virus carryover potentials.

In conclusion, we showed that PPE fabrics can mediate transmission of infectious viruses. Virus carryover potential varies among different fabrics, reflecting the fluid-repellency but not barrier efficiency. Body fluid repellency is measurable by biological and surface technologies and may provide a preferable index for the selection and improvement of PPE.

## Author Contributions

MM, SA, KS, and YU: study conception and design. NS, TK, YU, and KM: provision of materials and analysis tools. IK, HK, and FT: acquisition of data. IK, HK, FT, and MM: analysis and interpretation of data. IK: drafting of manuscript. FT and MM: critical revision.

### Conflict of Interest Statement

The authors declare that the research was conducted in the absence of any commercial or financial relationships that could be construed as a potential conflict of interest.
