# Supplementary material for: Potential Risk of Virus Carryover by Fabrics of Personal Protective Gowns
Source: Front Public Health. 2019 May 22;7:121. doi: 10.3389/fpubh.2019.00121 (PMC6538680; doi:10.3389/fpubh.2019.00121)
Supplement: Supplementary file 1 [file Presentation_1.pdf]

Supplementary Material

Potential Risk of Virus Carryover by Fabrics of Personal Protective Gowns

Iyoko Katoh[1*], Fuminori Tanabe[2], Hirotake Kasai[2], Kohji Moriishi[2], Noriko Shimasaki[3], Katsuaki Shinohara[4], Yukiko Uchida[5], Tomoko Koshiba[6], Soichi Arakawa[7], and Michiko Morimoto[8]

[1] Oral Health Science Research Center, Kanagawa Dental University, Yokosuka, Japan

[2] Faculty of Medicine, University of Yamanashi, Chuo, Japan

[3] Influenza Virus Research Center, National Institute of Infectious Diseases, Tokyo, Japan

[4] Division of Biosafety Control and Research, National Institute of Infectious Diseases, Tokyo, Japan

[5] Faculty of Health and Welfare, Takasaki University of Health and Welfare, Takasaki, Japan

[6] Faculty of Fashion Science, Bunka Gakuen University, Tokyo, Japan

[7] Sanda City Hospital, Sanda, Japan

[8] Faculty of Health and Welfare Science, Okayama Prefectural University, Soja, Japan

Correspondence: Iyoko Katoh: katoh.iyoko@kdu.ac.jp

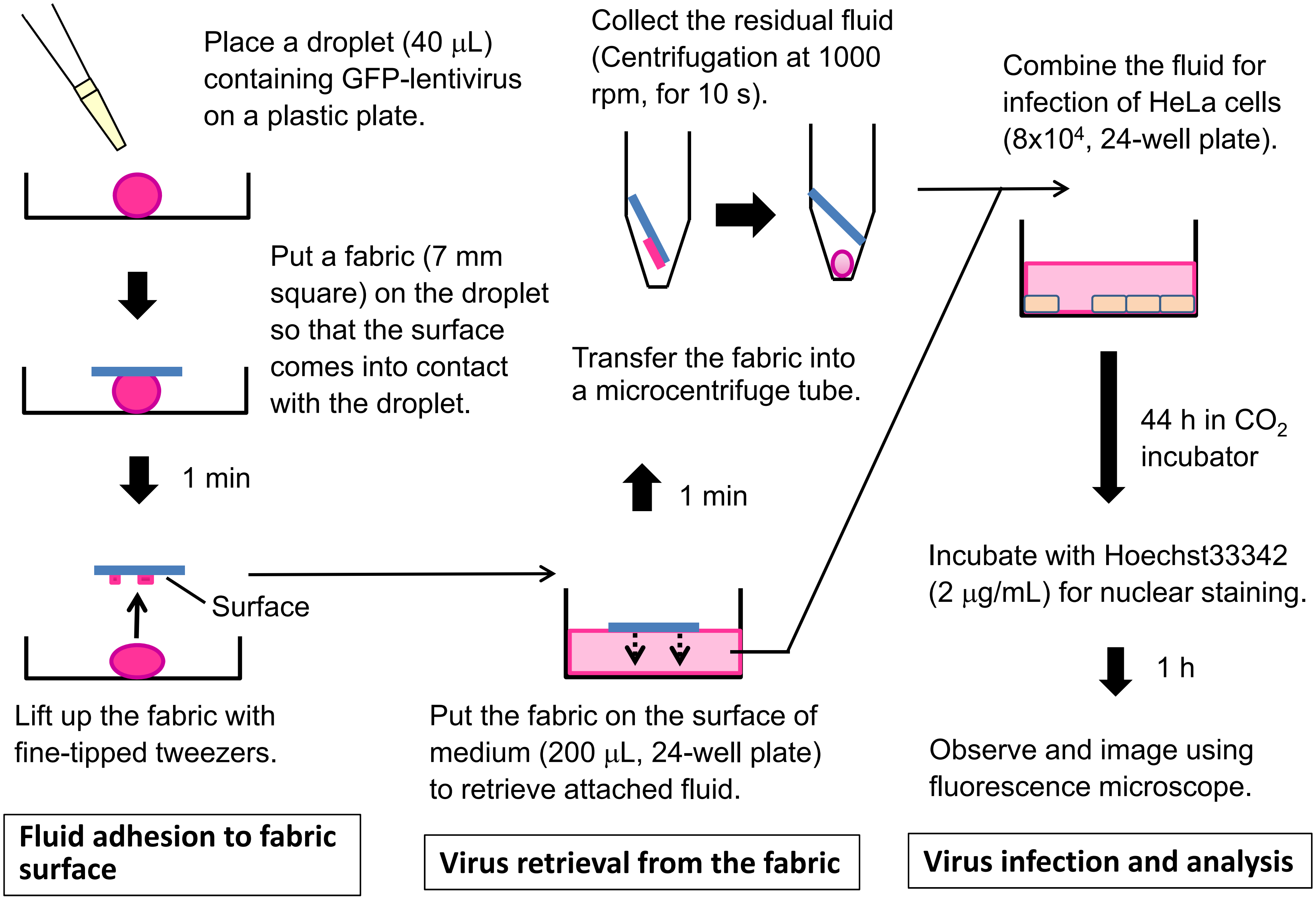

Supplementary Figure 1. Procedure of the virus carryover experiment.
